# Radiomics from Various Tumour Volume Sizes for Prognosis Prediction of Head and Neck Squamous Cell Carcinoma: A Voted Ensemble Machine Learning Approach

**DOI:** 10.3390/life12091380

**Published:** 2022-09-05

**Authors:** Fuk-Hay Tang, Eva-Yi-Wah Cheung, Hiu-Lam Wong, Chun-Ming Yuen, Man-Hei Yu, Pui-Ching Ho

**Affiliations:** School of Medical and Health Sciences, Tung Wah College, Hong Kong, China

**Keywords:** radiomics, radiotherapy, artificial intelligence (AI), HNSCC, GTV, PTV, prognosis prediction, head and neck cancer, TCIA, machine learning

## Abstract

Background: Traditionally, cancer prognosis was determined by tumours size, lymph node spread and presence of metastasis (TNM staging). Radiomics of tumour volume has recently been used for prognosis prediction. In the present study, we evaluated the effect of various sizes of tumour volume. A voted ensemble approach with a combination of multiple machine learning algorithms is proposed for prognosis prediction for head and neck squamous cell carcinoma (HNSCC). Methods: A total of 215 HNSCC CT image sets with radiotherapy structure sets were acquired from The Cancer Imaging Archive (TCIA). Six tumour volumes, including gross tumour volume (GTV), diminished GTV, extended GTV, planning target volume (PTV), diminished PTV and extended PTV were delineated. The extracted radiomics features were analysed by decision tree, random forest, extreme boost, support vector machine and generalized linear algorithms. A voted ensemble machine learning (VEML) model that optimizes the above algorithms was used. The receiver operating characteristic area under the curve (ROC-AUC) were used to compare the performance of machine learning methods, including accuracy, sensitivity and specificity. Results: The VEML model demonstrated good prognosis prediction ability for all sizes of tumour volumes with reference to GTV and PTV with high accuracy of up to 88.3%, sensitivity of up to 79.9% and specificity of up to 96.6%. There was no significant difference between the various target volumes for the prognostic prediction of HNSCC patients (chi-square test, *p* > 0.05). Conclusions: Our study demonstrates that the proposed VEML model can accurately predict the prognosis of HNSCC patients using radiomics features from various tumour volumes.

## 1. Introduction

Head and neck cancer (HNC) is a heterogeneous disorder of neoplasm, predominantly head and neck squamous cell carcinoma (HNSCC), which originates in the squamous epithelium lining the mucosal layer of the head and neck region. HNSCC may involve the nasal cavity, paranasal sinuses, nasopharynx, lips, oral cavity, tonsils, parotid glands and larynx [1]. HNC cancer accounted for approximately 900,000 cases globally in 2021, leading to more than 400,000 patient deaths annually [2]. In Hong Kong, HNC is one of the major causes of morbidity and mortality, with 1600 new cases being diagnosed annually [3]. Approximately 74% of treatment for HNC includes surgery and postoperative radiotherapy (RT) with concurrent chemotherapy [4]. The 5-year relative survival at diagnosis in the United States between 2010 and 2016 was approximately 80% for early-stage local disease and below 50% for late-stage disease [5]. In all cases of HNC, failure in locoregional control is attributed as the major cause of treatment failure and can result in a higher chance of distant metastasis and, thus, poor 5-year survival. It appears that more precise RT planning may help to improve 5-year survival in HNSCC.

Artificial Intelligence (AI) and machine learning are subfields of computer science involving algorithms to execute tasks, particularly those that require human-level intelligence [6]. In recent years, an increasing number of quantitative imaging studies about the applications of AI in HNC have been conducted to provide more critical information to support the clinicians’ decision. Previous studies showed that AI can help to characterize different types of dementia [7] and cancers [8] and facilitate the diagnosis of HNC [9]. Rahman et al. [10] achieved an accuracy of 100% in the textural classification of oral histology images to differentiate oral squamous cell carcinoma from normal images using AI with a support vector algorithm. Lee et al. [11] improved the diagnosis of cervical lymph node metastasis in HNC and achieved excellent accuracy, sensitivity and specificity. With respect to machine learning algorithms, decision tree (DT), random forest (RF), extreme boost (EB), support vector machine (SVM) and generalized linear (Linear) models are common algorithms employed in prognosis prediction and computer-aided diagnosis. A recent study suggested that using multiple algorithms may achieve higher accuracy when compared to single algorithm in machine learning [12]. Initiatives have been undertake to use radiomics features from various imaging modalities, such as CT, MRI and PET, to predict the 5-year survival of HNC patients in conjunction with bioimaging characteristics, such as programmed death ligand 1 (PD-L1) expression levels and human papillomavirus (HPV) status [13,14,15,16]. Bogowicz et al. [13] and Huang et al. [14] used CT radiomics to predict prognosis based on heterogeneity of tumour density in head and neck squamous cell carcinoma (HNSCC) and achieved an AUC of more than 0.75.

Gross tumour volume (GTV) refers to the volume that comprises the primary tumour and any diseased gross lymph nodes, whereas clinical target volume (CTV) includes the potential microscopic spread with an extension from the GTV. Planning target volume (PTV) refers to the additional margin added to GTV to account for physiologic organ mobility during therapy and setup uncertainties [17]. CT images for radiotherapy planning contain contours for target volume and organs at risks. With respect to target volumes, the gross tumour volume (GTV), clinical target volume (CTV) and planning target volume (PTV) are commonly used. Furthermore, GTV is the gross palpable extent and the location of the malignant growth. It consists of primary gross tumour volume (GTVp), which covers the primary tumour; the lymphadenopathy gross tumour volume (GTVn), which covers the metastatic lymph nodes; and the metastatic gross tumour volume (GTVm), which covers metastases to other organs. Due to the high concentration of malignant cells in GTV, a sufficient dose should be prescribed to achieve good local control in radical treatment. CTV is the additional volume added to GTV to cover subclinical spread of malignant cells. With respect to characteristics of fractionation of radiation therapy, the target volume captured in CT image is static, but the tumour moves or varies in size inter-fractionally in real-world situations. In addition, various treatment techniques require different tumour margins to ensure an adequate dose will be delivered to the CTV in various patient positions and beam geometries. To take the above challenges into considerations, a static PTV was defined, which covers CTV with additional volume to accommodate the variation in shape, size and position of the target volume, together with the treatment techniques employed with consideration for beam arrangements and patient positioning [17]. Our previous studies suggested that using radiomics with GTV and PTV can accurately predict prognosis [18]. However, a study is needed to address the knowledge gap in predicting the effect of different sizes and shapes of tumours for treatment effects. The tumour volumes (GTV and PTV) required for radiomics feature extraction were delineated by clinical oncologists and are only available in radiotherapy planning CT images but not in diagnostic CT images. Thus, the application of prognosis prediction was limited to patients who received radiotherapy, with tumour volume delineated. Furthermore, our results demonstrate that prognosis predication capabilities were similar using radiomics of PTV and GTV, with an accuracy of 74.3% and an AUC of 94.7%. If the size of tumour volume allows for certain extent of uncertainty in prognosis prediction, the delineation of tumour volume for radiomics feature extraction is less challenging and can be achieved by non-clinical staff for diagnostic CT. Thus, the application of radiomics for prognosis prediction can be extended to patients who received immunotherapy, target therapy and chemotherapy.

In this study, we aimed to determine the effects of various tumour volumes for radiomics features retrieval using different and combined ML algorithms to predict prognosis for head and neck squamous cell carcinoma (HNSCC) patients.

## 2. Materials and Methods

### 2.1. Patient Data

Planning CT images were retrieved from The Cancer Imaging Archive (TCIA). TCIA is a service that de-identifies and hosts a massive publicly accessible and reliable archive of collections of cancer medical images, along with patient outcomes, treatment information and genomics in a wide range of imaging modalities, such as MRI, CT, etc., under the management by the Frederick National Laboratory for Cancer Research and sponsorship of the Cancer Imaging Program (CIP), which is part of the US National Cancer Institute (NCI) [19]. The collections are reviewed and approved by the TCIA Advisory Group to ensure the integrity of the data, making them a reliable source for the present study. The Advisory Group is composed of staff from the National Cancer Institute (NCI) and the Frederick National Laboratory for Cancer Research (FNLCR), who are experts in cancer imaging, informatics and related technologies.

The dataset comprises head and neck squamous cell carcinoma (HNSCC) images. Two hundred and fifteen patients were treated for HNSCC with curative-intent radiation therapy (RT) from 2003 to 2013 [20,21,22]. Diseases were staged by the American Joint Committee on Cancer using the 7th Edition of the TNM system (AJCC-TMN) [23]. The treatment modalities included RT alone (66–70 Gy), concurrent chemo–RT (66–72 Gy) with a combination of 2–3 chemo drugs and postoperative RT or chemo–RT. The treatment modalities were decided based on the stage and the site of tumour. The data included planning CT images of the selected patients with RT structures, such as GTV and PTV. Clinical data, including the staging, dosage and 5-year survival, were also collected. However, there were 80 patients with incomplete data (either imaging data or part of the clinical data were not available), were excluded from this study. Ultimately, a total of 135 patients were included in this study.

### 2.2. Variation of Target Volumes

We investigated six target volumes: (1) GTV, (2) diminished GTV (GTV-2mm), (3) extended GTV (GTV+2mm), (4) PTV, (5) diminished PTV (PTV-2mm) and (6) extended PTV (PTV+2mm). GTV and PTV were delineated by clinical oncologists. the other four volumes (i.e., diminished GTV (GTV-2mm), extended GTV (GTV+2mm), diminished PTV (PTV-2mm) and extended PTV (PTV+2mm) were delineated by the study investigators using the Varian Eclipse treatment planning system version 15.6 (Varian, Palo Alto, CA, USA). All tumour volumes were checked by a certified medical dosimetrist.

### 2.3. Study Workflow

**Figure 1:**
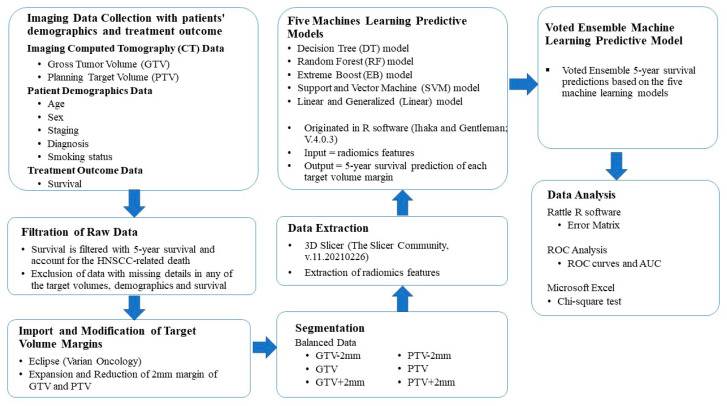
Diagram outlining the procedure of the present study. The definitions for each machine learning algorithm are attached in Appendix A.

The workflow of this study consisted of data collection, filtration of raw data, image importation and delineation of various target volumes, segmentation, data extraction, analysis by various machine learning algorithms, a voted ensemble machine learning model and data analysis (see Figure 1).

### 2.4. Feature Extraction

Radiomics features were extracted using a 3D slicer (The Slicer Community; V.4.11.20210226) with the PyRadiomics extension (Computational Imaging and Bioinformatics Lab, Harvard Medical School) [24]. A total of 107 radiomics features were extracted from each planning CT image, including tumour shape (n = 14), grey-level dependence matrix (n = 14), grey-level co-occurrence matrix (n = 24), first-order statistics (n = 18), grey-level run length matrix (n = 16), grey-level size zone matrix (n = 16), and neighbouring grey tone difference matrix features (n = 5). Finally, the numeric values of these features were input into the machine learning algorithms compiled by R (Ihaka and Gentleman; v. 4.0.3) with rattle and RGtk2 packages for prognosis prediction of HNSCC patients. 

### 2.5. Test to Avoid Overfitting

In order to avoid overfitting due to unbalanced sample data (i.e., bias in normal or abnormal cases), a balanced sample with an equal number of treatment outcomes, i.e., survived in 5 years or not survived in 5 years, was selected randomly to conduct an overfitting test (see Table 1).

### 2.6. Machine Learning Algorithms

Machine learning algorithms including decision tree (DT) algorithm, random forest (RF) algorithm, extreme boost (EB) algorithm, support vector machine (SVM) algorithm and generalized linear (Linear) algorithm were employed in this study. These are common machine learning algorithms, so our results can be compared directly with those of other studies. The radiomics data were trained and tested against the treatment outcomes, which were randomly split into three independent cohorts in each machine learning algorithm, with 70% data for the training cohort, 15% data for the validation cohort and 15% data for the testing cohort.

The predictive outcome was presented as a binary output, i.e. decimal value ranging between 0 and 1 (with 0 and 1 inclusive). Decimal values of less than 0.5 indicate a model prediction of patient survived after 5 years or died of non-HNSCC-related diseases. Decimal values equal to or greater than 0.5 indicate a model prediction of patient died within 5 years after diagnosis. 

### 2.7. Voted Ensemble Machine Learning Model

Various machine learning algorithms have their strengths and weaknesses. Our preliminary study indicated that the decision tree algorithm was more sensitive to variation in target volumes, but it yielded low sensitivity and specificity. On the other hand, random forest yielded good accuracy, sensitivity and specificity, although it required long processing time and considerable hardware processing power.

**Figure 2:**
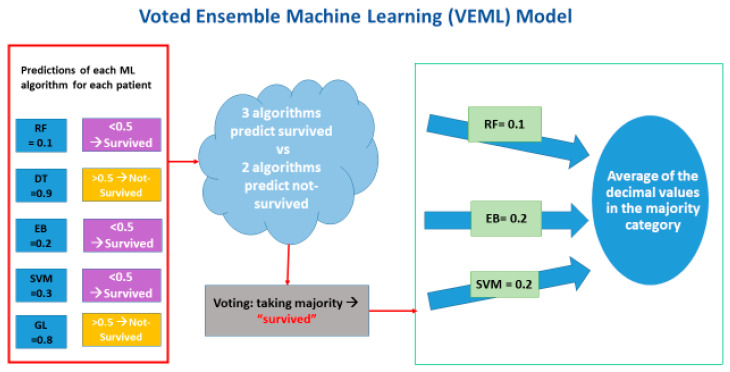
Schematic diagram of the voted ensemble machine learning model.

The ensemble method involves an election among multiple machine learning algorithms to obtain improved predictive performance relative any of the constituent learning algorithms alone. We adopted an ensemble machine learning concept, called the voted ensemble machine learning (VEML) model, to integrate the results derived from multiple machine learning algorithms. A previous study suggested that the VEML model can achieve better predictive performance than a single algorithm by voting on the outputs by all algorithms in a bagging process [25]. The prediction results from each machine learning algorithms, i.e., decision tree (DT), random forest (RF), extreme boost (EB), support vector machine (SVM) and generalized linear (Linear) algorithms, were gathered and fit into the voted ensemble machine learning (VEML) model. An average of prediction results was calculated based on the major outcome, i.e., survived or not survived. The uniqueness of our VEML model is that it integrates the 5 most common ML algorithms. The predictive result was voted on by a majority prediction result (see Figure 2).

### 2.8. Data Analysis

The receiver operating characteristic (ROC) curve was used to illustrate the prognostic ability of a binary classifier (survived or not survived) and to analyse the performance of the machine learning algorithms. Area under the curve (AUC), accuracy, sensitivity and specificity were used as evaluation metrics. ROCkit (University of Chicago, 1995) was used to generate the ROC curve and AUC. Chi-square tests were used to determine whether there were statistically significant differences between the investigated machine learning algorithms.

## 3. Results

### 3.1. Cohort Demographics

The HNSCC dataset comprises 215 patients, of which 80 patients were excluded from this study due to incomplete imaging or clinical data. As a result, 135 patients were included in this study. The demographics of the study cohort are shown in Table 2.

### 3.2. Prognosis Prediction of Five Machine Learning Algorithms and VEML Model

For 92 patients with GTV-varied radiomics features and 98 patients with PTV-varied radiomics features, the machine learning algorithms with balanced data showed a wide range of AUC in terms of 5-year survival predictions. The EB algorithm and the RF algorithm demonstrated excellent 5-year survival predictions for all target volumes with AUCs of 0.9 or higher. The other three algorithms, including DT, SVM and Linear, demonstrated fair 5-year survival predictions in all target volumes, ranging from 0.760 to 0.861 in for DT, from 0.823 to 0.882 for SVM and from 0.757 to 0.803 in for Linear. Detailed results are shown in Table 3.

### 3.3. Prediction Performance of the Five Machine Learning Models Using Radiomics Features of Various Target Volumes

**Figure 3:**
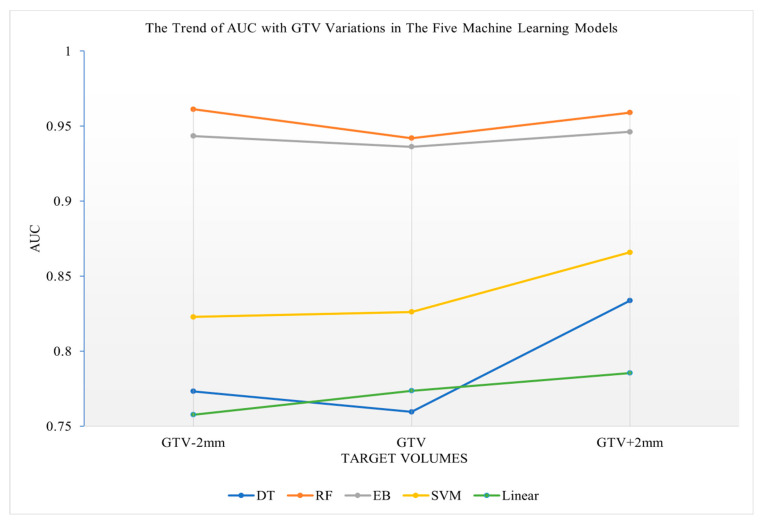
Trend of AUC relative to GTV variations in the five machine learning algorithms.

The RF algorithm and the EB algorithm, compared to the DT algorithm, the SVM algorithm and the Linear algorithm, showed lower sensitivity to target volume variations in terms of 5-year survival prediction (see Figure 3). The curves were flat with minor variations among all target volume variations in both the RF and the EB algorithms, whereas the curves varied more sensitively to the target volume variations in the DT algorithm, the SVM algorithm and the Linear algorithm. The DT algorithm was the most sensitive to target volume variations among the five investigated algorithms.

**Figure 4:**
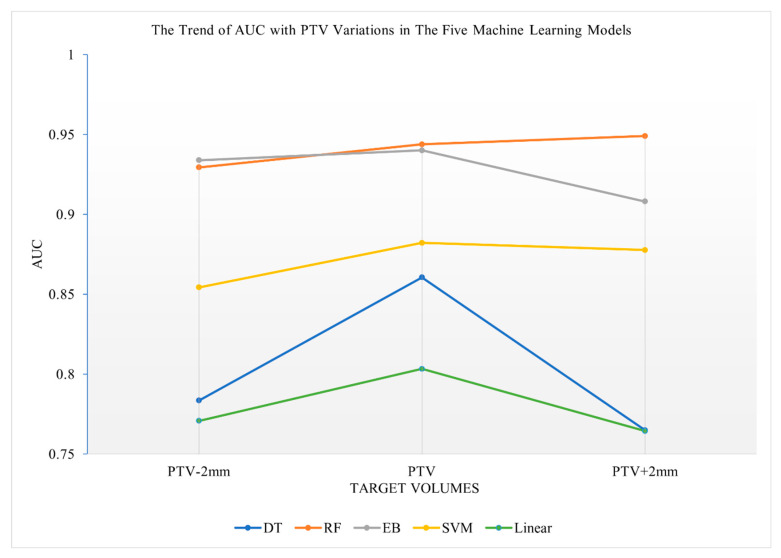
Trend of AUC relative to PTV variations of the five machine learning algorithms.

When the margins increased from PTV-2mm to PTV or from GTV to GTV+2mm, the curves of all five investigated algorithms showed an increase in AUC values, indicating improved 5-year survival prediction for the PTV or GTV, with a 2 mm expanded margin when compared to PTV-2mm or GTV (Figure 3 and Figure 4).

**Figure 5:**
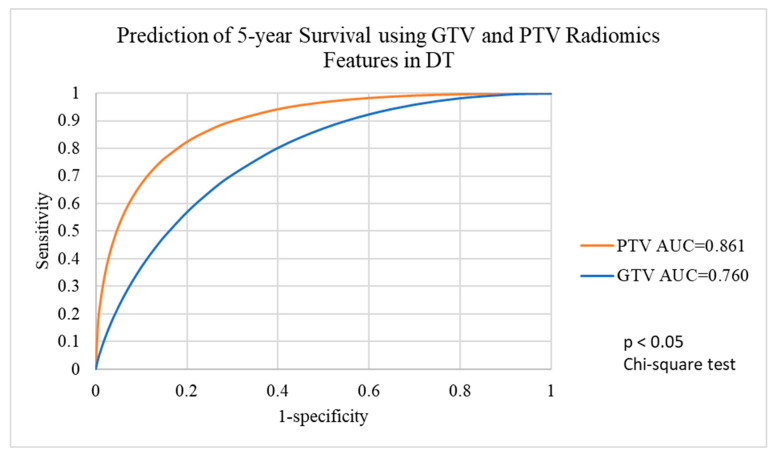
Prediction of 5-year survival using GTV and PTV radiomics features with the DT algorithm. GTV, gross tumour volume; PTV, planning target volume.

**Figure 6:**
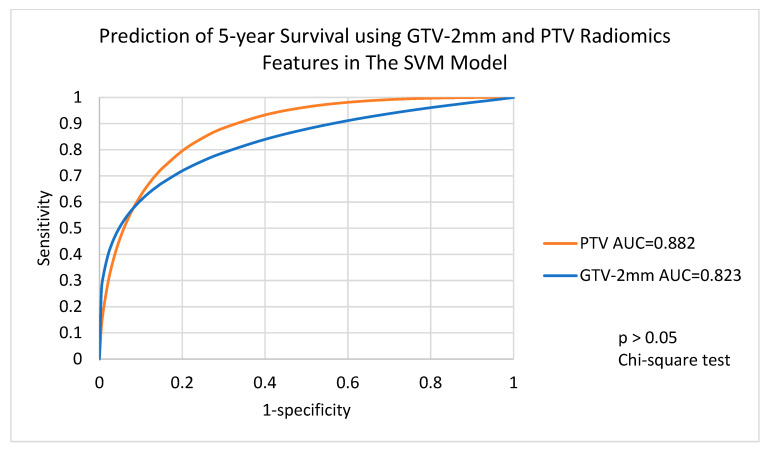
Prediction of 5-year survival using GTV-2mm and PTV radiomics features with the SVM algorithm. GTV-2mm, gross tumour volume 2mm margin; PTV, planning target volume.

**Figure 7:**
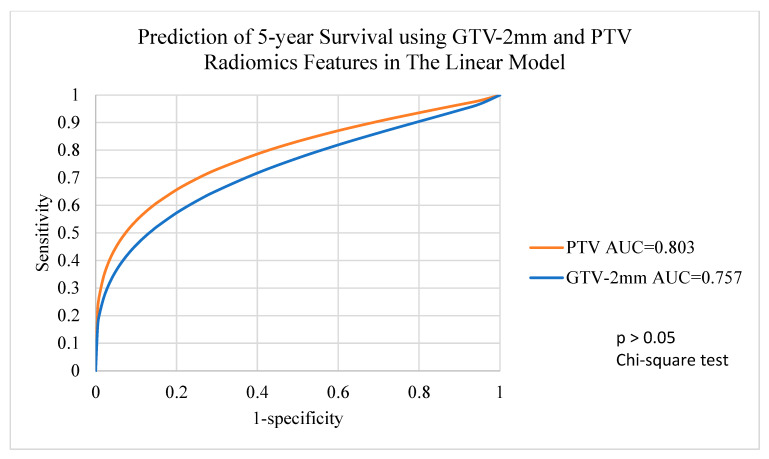
Prediction of 5-year survival using GTV-2mm and PTV radiomics features with the Linear algorithm. GTV-2mm, gross tumour volume 2mm margin; PTV, planning target volume.

ROC analysis (Figure 5) indicated that there was a significant difference between using GTV and PTV radiomics features with the DT algorithm to predict 5-year survival (chi-square test, *p* < 0.05), whereas the SVM algorithm (Figure 6) and the Linear algorithm (Figure 7) did not show a significant difference in 5-year survival prediction in the ROC analysis.

The sensitivity of predictions using the GTV radiomics features was relatively low (50.0%) with the DT algorithm. The DT algorithm also had the lowest average accuracy of 77.0% (compared with other four algorithms, with average accuracies ranging from 77.5% to 88.7%), the lowest average sensitivity of 65.0% (compared with other four algorithms, with average sensitivities ranging from 71.4% to 83.5%) and the second lowest average specificity of 83.8% (compared with other four algorithms, with average specificities ranging from 83.6% to 96.6%) (Table 3). Given that there were discrepancies among the investigated algorithms in terms of accuracy, sensitivity and specificity, the voted ensemble machine learning model was proposed to balance out the bias and variances of the five machine learning algorithms.

### 3.4. Performance of Voted Ensemble Machine Learning Model

The voted ensemble machine learning model achieved an AUC more than 0.920 for all target volumes, indicating good prognosis predictions for all target volumes. Details are shown in Table 3.

**Figure 8:**
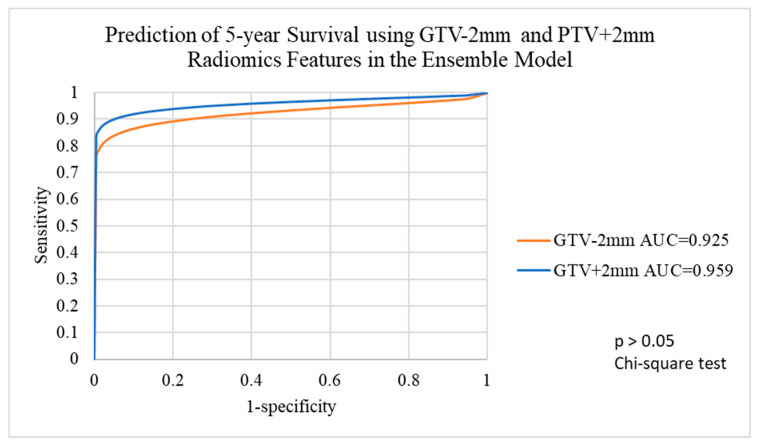
Prediction of 5-year survival using GTV-2mm and GTV+2mm radiomics features with the voted ensemble machine learning model. GTV-2mm, GTV-2 mm margin; GTV+2mm, GTV+2mm margin.

ROC analysis indicated that there was no significant difference between using GTV-2mm and GTV+2mm radiomics features, which showed the greatest difference in AUC in predicting 5-year survival with the voted ensemble machine learning model (chi-square test, *p* > 0.05) (Figure 8).

The voted ensemble machine learning model showed a higher average accuracy, reaching 88.3% (compared to 87.6% with EB, 77.0% with DT, 77.5% with SVM and 85.6% with Linear), a higher average sensitivity of 79.9% (compared to 78.5% with EB, 65.0% with DT and 71.4% with SVM) and a high specificity of 96.6% (compared to 83.8% with DT, 83.6% with SVM and 87.7% with Linear) (Table 3).

## 4. Discussion

In this study, we evaluated the performance of five algorithms and the VEML model with respect to 5-year survival prediction in HNSCC patients using the radiomics features of various target volumes. We noted that although different models showed slight changes in prediction of prognosis, no significant differences were demonstrated using chi-square test, except with the DT algorithm.

### 4.1. The Value of the VEML Model

In this study, although the GTV and PTV radiomic features used with the DT model were exhibited a statistically significant difference in terms of prediction of 5-year survival of HNSCC patients, the sensitivity of using the GTV radiomic features was relatively low, at 50.0%. The other four machine learning algorithms did not indicate a significant difference between the use of GTV and PTV radiomic features for the prediction of 5-year survival. In terms of average accuracy, sensitivity and specificity, the VEML model slightly outperformed the majority of the five machine learning algorithms, providing predictions by combining predictions from the five machine learning algorithms. The application of the VEML model can balance the strengths and weaknesses of different machine learning algorithms, by implementing the voted ensemble learning concept, to reduce the effects of single machine learning algorithm and minimize the bias and variations in prognosis predictions. Therefore, the VEML model can improve the stability of the overall predictive performance for HNSCC 5-year survival.

### 4.2. Application of the VEML Model to Supplement TNM Staging for Prognosis Prediction

The TNM staging system is the traditional method of predicting 5-year survival outcomes in cancer patients. The TNM staging method can be used to evaluate the therapeutic options available to HNSCC patients based on tumour size, local penetration, histology, lymphatic dissemination and metastatic status. Although the TNM staging approach is regarded as a reliable tool for RT decision making, it requires clinical results, which can be invasive for patients, such as biopsy, along with imaging scans of the results.

In comparison to predictive models proposed in previous studies based on the TNM staging system and clinical characteristics, the VEML model proposed in this study uses radiomics features of various target volumes to achieve an overall high AUC with excellent accuracy, sensitivity and specificity. For example, Bryce et al. [26] conducted a similar study using the TNM system for prognostic prediction of HNSCC patients, with clinical parameters such as tumour size, nodal disseminations, stage at diagnosis and resectability employed to build a 2-year survival predictive model for HNSCC patients. Our VEML model achieved a high overall AUC of 0.920, with a high sensitivity of 79.9% and specificity of 96.6% among all target volumes when compared to their model, which achieved an AUC of 0.670, with 72.0% and 70.0% in specificity and sensitivity, respectively, in terms of 5-year survival prediction of HNSCC patients [26].

A similar study used a gene-related random forest model to forecast 5-year survival of HNSCC patients, with an AUC of 0.74, showing a lower predictive performance when using gene-related clinical data compared to radiomics features of target volumes as in our VEML model [27]. This is also supported by a similar study with an AUC of 0.893 using the integration of novel immune-related genomic biomarkers of HNSCC to predict 5-year overall survival [28]. Our VEML model achieved a better predictive performance using the radiomics features of various target volumes, with an AUC of 0.920, when compared to the traditional predictive model based on biomarkers for 5-year survival prediction in HNSCC patients.

Luo et al. [29] developed a model to analyse radiomics features, as well as clinical and dosimetric parameters, through a combination of multiple algorithms to predict 5-year survival in lung cancer patients. The model performed significantly better than that using a single machine learning algorithm. Their result is consistent with the findings of our study, in which the VEML model based on radiomics features performed better than a single machine learning algorithm.

When compared to our previous study using a deep learning artificial network (DL-ANN) [18] using radiomics features of GTV and PTV to predict 5-year survival prediction for HNSCC patients, the VEML model achieved similar performance to the DL-ANN. In view of using PTV radiomics features, the VEML model achieved a higher AUC (0.947 vs. 0.925 with the DL-ANN), higher accuracy (88.8% vs. 77.7% with the DL-ANN) and higher specificity (93.9% vs. 72.0% with the DL-ANN) but lower sensitivity (81.6% vs. 95.6% with the DL-ANN). In view of using GTV radiomics features, the VEML model achieved a slightly lower AUC (0.927 vs. 0.946 with the DL-ANN), the same accuracy (85.9% vs. 85.9% with the DL-ANN) and higher specificity (97.8 vs. 86.4% with the DL-ANN) but lower sensitivity (73.9% vs. 84.2% with the DL-ANN). On the whole, both the proposed VEML model and the DL-ANN model showed promising results, with high accuracy, sensitivity and specificity. The VEML demonstrated good prediction performance with various target volumes, suggesting that the prediction performance could accommodate slight variations in the size of the target volume in retrieving the radiomics features to predict the 5-year survival of HNSCC patients.

We suggest that radiomics features are based on the distribution of pixel values inside the tumour rather than the actual size of the tumour, with slight variations in GTV and PTV having no effects on prognosis prediction, although PTV delineation for radiotherapy treatment should consider clinical implications, such as cancer recurrence and the radiation dose to nearby organs at risk.

### 4.3. Potential Development of the VEML Model

In this study, we used radiomics features with the VEML model for prognosis prediction. The model can be improved by incorporating other parameters for prognosis prediction. Bao et al. [30] demonstrated that combining radiomics and clinical parameters could achieve a higher AUC of 0.780 when compared to that with the use of radiomics features alone, with and AUC of 0.626. More predictive parameters, such as epidermal growth factor receptor, PD-L1 expression and HPV status, can be added into the model to improve the 5-year survival prediction [13,30]. Incorporating more radiomics features from other imaging modalities, such as PET/CT and MRI, may also improve the predication capabilities of the model [9].

### 4.4. Advantages of this Work Compared with Other Related Work

Our study using the VEML method has the advantage of being more stable and less error-prone when compared with other related works. Furthermore, the present study is the first to address the effect of delineating various tumour volume sizes for prognostic prediction based on radiomic features, which may reduce observer variations for tumour volume delineation. This forms a more objective method than those used in other studies.

### 4.5. Study Limitations

Our analysis was based on a dataset from a single centre. The proposed model should be validated in an independent cohort to confirm the application. The sample size in the present study was also small after excluding some subjects due to incomplete data. A previous study [31] indicated that accuracy varied with sample size and depended on sample heterogeneity. Further study is suggested with a larger sample size. Furthermore, only radiomics features were used as prediction parameter in this study. The inclusion of other prediction parameters, such as demographics data, dosimetric data from radiotherapy treatment and radiomics features from multiple imaging modalities, may help to improve the model performance.

## 5. Conclusions

In this study, we evaluated the effect of various target volumes on the 5-year survival prognosis of the HNSCC patients using radiomics features. The VEML model yielded promising 5-year survival predictions based on radiomics features of various target volumes, demonstrating a high average accuracy (88.3%), sensitivity (79.9%) and specificity (96.6%), as well as a high predictive performance in 5-year survival in terms of AUC. The VEML model has the benefits of being stable and objective and can eventually be used as a reliable predictive tool for clinical decision making when clinical parameters and multiple imaging modalities are included in the model.

## Figures and Tables

**Table 1:** Balanced sample size with various tumour volumes.

Target Volumes	GTV-2mm	GTV	GTV+2mm	PTV-2mm	PTV	PTV+2mm	PTV+4mm
Sample Size	92	92	92	98	98	98	98
Balanced sample	46 not-survived; 46 survived	46 not-survived; 46 survived	46 not-survived; 46 survived	46 not-survived; 46 survived	49 not-survived; 49 survived	49 not-survived; 49 survived	49 not-survived; 49 survived

**Table 2:** Patient demographics, tumour characteristics and clinical data.

Patient and Tumour Characteristics (All n = 135)	Data
Age range (years)	34–91
**Sex**	
Female	20
Male	115
**Staging**	
Stage I	4
Stage II	2
Stage III	17
Stage IVA	102
Stage IVB	10
**Diagnosis**	
Ca Base of Tongue	48
Ca Tonsil	41
Ca others	46
**Smoking status**	
Smoker	86
Non-smoker	49

**Table 3:** A Summary of accuracy, sensitivity and specificity of prognosis prediction with various target volumes in six machine learning models.

	Machine Learning Model	GTV -2mm	GTV	GTV +2mm	PTV-2mm	PTV	PTV +2mm	Average
**AUC**	RF	0.961	0.942	0.959	0.929	0.944	0.949	-
	EB	0.943	0.936	0.946	0.934	0.940	0.908	-
	DT	0.773	0.760	0.834	0.783	0.861	0.765	-
	SVM	0.823	0.826	0.866	0.854	0.882	0.878	-
	Linear	0.757	0.774	0.785	0.771	0.803	0.764	-
	VEMLM	0.925	0.927	0.959	0.927	0.947	0.956	-
**Accuracy**	RF	0.880	0.891	0.891	0.867	0.909	0.887	0.887
	EB	0.870	0.880	0.880	0.867	0.867	0.888	0.876
	DT	0.750	0.739	0.782	0.755	0.806	0.786	0.770
	SVM	0.739	0.761	0.750	0.785	0.806	0.806	0.775
	Linear	0.859	0.848	0.859	0.847	0.867	0.857	0.856
	VEMLM	0.880	0.859	0.902	0.878	0.888	0.888	0.883
**Sensitivity**	RF	0.761	0.783	0.804	0.816	0.857	0.837	0.810
	EB	0.739	0.783	0.761	0.796	0.816	0.816	0.785
	DT	0.609	0.500	0.587	0.612	0.878	0.714	0.650
	SVM	0.587	0.652	0.717	0.735	0.776	0.816	0.714
	Linear	0.826	0.848	0.804	0.857	0.796	0.878	0.835
	VEMLM	0.761	0.739	0.804	0.816	0.837	0.837	0.799
**Specificity**	RF	1.000	1.000	0.978	0.918	0.959	0.939	0.966
	EB	1.000	0.978	1.000	0.938	0.918	0.959	0.966
	DT	0.581	0.978	0.978	0.898	0.735	0.857	0.838
	SVM	0.891	0.870	0.783	0.837	0.837	0.796	0.836
	Linear	0.891	0.848	0.913	0.837	0.939	0.837	0.877
	VEMLM	1.000	0.978	1.000	0.939	0.939	0.939	0.966

## Data Availability

Publicly available datasets were analysed in this study. These data can be found at: https://wiki.cancerimagingarchive.net/display/Public/HNSCC#41518000036220c66a5a436f90e4a0b54367bfae (accessed on 22 January 2022). Data used in preparation of this article were obtained from The Cancer Imaging Archive (TCIA): Maintaining and Operating a Public Information Repository.

## Appendix A. Definitions of Machine Learning Models [32]

The decision tree (DT) model used a conditional tree algorithm as a recursive partitioning approach for data mining but was subject to the weakness of bias and variances.

The random forest (RF) model comprised a collection of unpruned decision trees with available variable subsets, exhibiting less bias and variances than a single decision tree.

The extreme boost (EB) model used a weight with each observation in the dataset and built a series of models of decision trees by boosting the weight to the model that incorrectly classified the observation.

The support vector machine (SVM) identified data at the boundaries between classes and identified the line that separated the classes in prediction.

The linear and generalized linear (Linear) model fitted a statistical model to data for a regression model in prediction.
